# Effect of COVID‐19 infection on sex hormone levels in hospitalized patients: A prospective longitudinal study in Iran

**DOI:** 10.1002/hsr2.1011

**Published:** 2022-12-24

**Authors:** Poorandokht Afshari, Mehrnoosh Zakerkish, Parvin Abedi, Maryam Beheshtinasab, Elham Maraghi, Hossein Meghdadi

**Affiliations:** ^1^ Midwifery Department Reproductive Health Promotion Research Center, Ahvaz Jundishapur University of Medical Sciences Ahvaz Iran; ^2^ Medicine School, Ahvaz Jundishapur University of Medical Sciences Ahvaz Iran; ^3^ Midwifery Department Menopause Andropause Research Center, Ahvaz Jundishapur University of Medical Sciences Ahvaz Iran; ^4^ Reproductive Health Promotion Research Center, Ahvaz Jundishapur University of Medical Sciences Ahvaz Iran; ^5^ Biostatistics Department Ahvaz Jundishapur University of Medical Sciences Ahvaz Iran; ^6^ Ahvaz Jundishapur University of Medical Sciences Ahvaz Iran

**Keywords:** COVID‐19, estradiol, follicle stimulating hormone, luteinizing hormone, progesterone

## Abstract

**Introduction:**

This study aimed to evaluate the levels of sex hormones in patients with COVID‐19 in Ahvaz, Iran.

**Methods:**

A prospective longitudinal study was conducted at Razi hospital, Ahvaz, Iran, from July 2020 to Febuary 2021. The levels of sex hormones including estradiol, progesterone, luteinizing hormone (LH), follicle stimulating hormone (FSH), and total and free testosterone were measured in 162 patients with COVID‐19 infection during hospitalization and 1 month after discharge. A demographic questionnaire and a checklist were used to collect the data. Mann−Whitney *U* test, *χ* ^2^ test, Fisher's exact test, Wilcoxon test, and logistic regression were used to analyze the data.

**Results:**

Sex hormones were assessed in 162 patients at baseline; however, a month after discharge, only 69 patients provided consent for assessment, and 9 had passed away. The estradiol level was 407.70 ± 623.37 and 213.78 ± 407.17 pg/ml in female patients with severe and moderate diseases at baseline, respectively which reduced to 195.33 ± 380.04 and 58.20 ± 39.45 pg/ml after discharge (*p* = 0.011 and *p* = 0.001). The alteration in the levels of progesterone, LH, and FSH were not significant.

The level of LH in both groups of male patients with severe (6.64 ± 2.91 IU) and moderate disease (6.42 ± 4.44 IU) was high, which reduced after discharge (4.16 ± 2.44 and 3.93 ± 3.15 IU, respectively), but this decrease was significant only in the patients with severe disease (*p* < 0.0001). The alteration of FSH and free testosterone were not significant. The level of testosterone was 1.19 ± 0.73 and 1.46 ± 1.22 ng/ml at baseline in patients with severe and moderate diseases which increased to 2.64 ± 1.25 ng/ml, *p* < 0.0001, and 2.54 ± 0.93 ng/ml, *p* = 0.001, respectively after discharge.

**Conclusion:**

Our findings showed that the level of estradiol in female patients increased significantly while the level of testosterone in male patients decreased during the active phase of infection. Due to the attrition of patients in the follow‐up period, more studies are needed to confirm these results.

## 1 INTRODUCTION

The SARS‐CoV‐2 or COVID‐19 viral infection that started in December 2019 from China, has now spread around the world, and around 240 million people have so far been infected with this virus.^1^ Countries such as Iran with delayed vaccination experienced the fifth surge of this infection, with cumulative confirmed deaths of 122,197 people.^2^ The negative effect of COVID‐19 on different aspects of physical health and well‐being and mental health (increasing anxiety and depression) have been proved in other studies.^3^, ^4^

There is limited evidence that women are less likely to contract COVID‐19 infection, and in case of infection, they show less severe disease symptoms such as thromboembolic events compared with men.^5^, ^6^ Data from some countries such as Italy which has experienced high rates of COVID‐19 infection have also shown that the mortality of men is more than that of women in all ages.^7^ The hypothalamus‐pituitary‐adrenal axis has been reported as the main target of COVID‐19 infection, and glucocorticoid has been suggested by almost all treatment regimens for the patients with moderate or severe forms of COVID‐19 infection.^8^ Since angiotensin‐converting enzyme‐2 (ACE2) receptor is expressed in endocrine organs and is also used by SARS‐CoV‐2 to attack the host cells, there is a hypothesis that endocrine organs and their hormones may be affected by this virus.^9^

The relationship between COVID‐19 infection and sex hormones has shown that female hormones such as estrogen and progesterone play a protective role in COVID‐19 infection in women. Mauvais‐Jarvis et al.,^10^ for instance, found that steroids 17β‐estradiol (E2) and progesterone have a protective role against COVID‐19 infection in women. In addition, Li et al.^11^ reported that sex hormone concentration and anti‐mullerian hormone in childbearing women did not change with COVID‐19 infection. However, other studies have shown decreased levels of male hormones in patients with COVID‐19 infection. Dhindsa et al.^12^ for example, studied 152 hospitalized patients and found that the patients had significantly low levels of testosterone. Also, the serum level of total testosterone in male patients with severe disease and mostly in those admitted to the intensive care unit (ICU) was significantly lower than that in patients with a mild infection.^13^

Given the limited number of studies on the relationship between sex hormones and COVID‐19 infection and their contradictory results, we aimed to investigate prospectively the level of sex hormones in male and female patients hospitalized in Ahvaz, Iran.

## 2 MATERIALS AND METHODS

### 2.1 Study design

This was a prospective longitudinal study in which 162 hospitalized male and female patients were assessed in terms of their sex hormones, upon admission to hospital and 1 month after discharge. The design of this study was approved by the Ethics Committee of Ahvaz Jundishapur University of Medical Sciences (Ref No: IR.AJUMS.REC.1399.541). All participants provided written informed consent before data collection.

### 2.2 Setting

All patients were recruited from Razi Hospital in Ahvaz, Iran. Razi Hospital is a university hospital with 200 beds that was designated to admission of patients with COVID‐19 infection from the beginning of the pandemic. Ahvaz is the capital of Khuzestan province in Iran and has a population of 1,244,000 according to the latest official census.^14^ Data collection was started in July 2020 and completed in February 2021 that was coincident with the second and third surges of COVID‐19 in Iran.^15^

### 2.3 Sample size

The necessary sample size for female participants was calculated based on a pilot study. Minimum sample size based on alteration of progesterone levels in infected women with COVID‐19 was calculated from the following formula:

(1) $$n=\frac{{\left(Z_{1-\frac{\alpha }{2}}\right)}^{2}s^{2}}{d^{2}}$$

Where *s* = 2.20, *d* = 0.5, and type 1 error of 5%. Then the total number of 75 was considered for female patients. For male participants, minimum sample size was calculated on the alteration of in free testosterone levels when individuals get infected with COVID‐19 calculated from above‐mentioned formula. Where *s* = 3.0, *d* = 0.65, and type 1 error of 5%. We aimed to recruit at least 82 men.

#### 2.3.1 Inclusion/exclusion criteria

All hospitalized patients (male and female) with confirmed COVID‐19 infection were recruited for this study. Pregnant or breastfeeding women, those with confirmed menopause, women with estrogen‐secreting tumors, and those on exogenous estrogen replacement were excluded from the study.

Diagnosis of COVID‐19 infection was confirmed using PCR. Admission to hospital meant that patients had moderate disease. Patients with moderate disease had blood oxygen more than 93%, and mild respiratory distress. A lung CT scan was requested for each patient with respiratory distress.

A severe disease was defined when the patient had a lung infiltration >50%, or if the level of blood oxygen was less than 93%, and a respiratory rate was more than 30 breaths/min. Severity of disease was confirmed by chest CT‐scan. All patients with these criteria were transferred to the ICU. The severity of disease was confirmed by an infectious disease specialist.

### 2.4 Outcomes

The changes in sex hormones in male and female hospitalized patients were outcomes of this study.

#### 2.4.1 Study variables

COVID‐19 infection was the independent variable and female and male sexual hormones were dependent variables.

### 2.5 Procedure

Five milliliter venous blood were drawn from hospitalized patients and placed into gel tubes, and transferred to a reference laboratory (Novin Lab). Then the blood samples were centrifuged for 10 min and the sera were separated in gamma tubes with a lead and stored in −20°C until the time of evaluation. The measurement technique was based on quantitative luminescence. For female patients, the levels of estradiol (pg/ml), progesterone (ng/ml), luteinizing hormone (LH) (IU), and follicle stimulating hormone (FSH) (IU) were measured, while assessment of male patients involved the level of testosterone (ng/dl), and free testosterone (ng/ml). Blood samples were collected by a trained midwife. One month after discharge from hospital, the patients were invited to attend the reference laboratory to collect new blood samples. Fasting was not necessary for blood sampling. The analyses on the second blood samples were done in the same laboratory and using the same technique as in the first round of evaluation. The normal levels of hormones in females were as follows: estradiol: follicular phase: 9−175 pg/ml, luteal phase: 44−196 pg/ml, preovulatory: 107−281 pg/ml. Progesterone: 0.05−9.24 ng/ml. LH: follicular phase: 1.1−11.6 IU, midcycle peak: 17−77 IU, luteal phase: 0.1−14.7 IU. FSH: follicular phase: 2.8−12 IU, midcycle peak: 5.8−21 IU, luteal phase: 1.2−9 IU. The normal levels of hormones for men were as follows: LH: 0.7−11.1 IU, FSH: 0.7−11.1 IU, testosterone: 2.27−10.30, free testosterone: 20−39 years: 9.2−34.6 ng/ml, 40−59 years: 6.1−30.3 ng/ml, ≥60 years: 6.1−27.9 ng/ml.

#### 2.5.1 Instruments

A demographic questionnaire and a checklist were used to collect the data. The demographic questionnaire included questions about age, sex, marital status, educational attainment, signs and symptoms of COVID‐19, and history of chronic diseases. The checklist was used to record the laboratory results.

### 2.6 Statistics

Results were presented as absolute frequencies and percentages for qualitative variables. For continuous variables results were reported as range and mean ± SD. Normality assumption was assessed using the Shapiro−Wilk test. Quantitative variables were compared with *t*‐test or Mann−Whitney *U*‐test, whenever appropriate. Wilcoxon's paired samples test was used to compare baseline and follow‐up measurements of hormones' levels. Categorical variables were compared using the *χ* ^2^ or Fisher's exact test. Multiple logistic regression models were used to examine whether there were relationships between baseline hormones levels and severity of infection adjusted for age and gender. The odds ratio was calculated and presented with a 95% confidence interval. Two‐sided *p* values less than 0.05 were considered statistically significant. Statistical analysis was performed using the statistical software SPSS 18.0.0. (SPSS Inc.).

## 3 RESULTS

In the initial assessment, 162 patients were assessed in terms of their serum levels of sex hormones. One month after discharge, 9 patients passed away (the mortality rate was 5.5%), and only 69 patients provided consent for vein puncture.

Table 1 shows the demographic characteristics of participants. Out of the 162 patients studied, 132 (81.4%) had moderate disease and 30 (18.5%) had severe disease who were transferred to ICU. The mean age of male and female participants was 43.04 ± 7.32 and 36.45 ± 5.31 years, respectively. Male patients 21 (26.9%) were more likely to have severe disease compared to women 9 (12%). The most prevalent chronic disease among participants was diabetes 11 (8.3%). Most patients 62 (38.3%) had all symptoms of the infection including fever, cough, headache, shortness of breath, and myalgia.

**Table 1:** Demographic characteristics of participants in the study

Variable	All participants (*N* = 162)	Moderate (*N* = 132)	Severe (*N* = 30)	*p* Value
Age (year); *Mean* ± *SD* (min−max)				
All	39.88 ± 7.04 (21−54)	39.75 ± 7.18	40.50 ± 6.50	0.60
Male	43.04 ± 7.32 (21−54)	43.04 ± 7.32	42.04 ± 6.67	0.558
Female	36.45 ± 5.31 (23−44)	36.45 ± 5.31	36.88 ± 4.59	0.935
Sex; *n* (%)				0.067
Female	75 (46.3)	66 (88)	9 (12)	
Male	87 (53.7)	66 (84.6)	21 (26.9)	
Occupation; *n* (%)				0.323
Unemployed	8 (4.9)	7 (5.3)	1 (3.3)	
Housewife (only for female)	63 (38.9)	55 (41.7)	8 (26.7)	
Manual worker	11 (6.8)	10 (7.6)	1 (3.3)	
Employed	39 (24.1)	30 (22.7)	9 (30.0)	
Self‐employed	41 (25.3)	30 (22.7)	11 (36.7)	
Education; *n* (%)				0.676
Primary	36 (22.2)	27 (20.5)	9 (30.0)	
High school	29 (17.9)	25 (18.9)	4 (13.3)	
Diploma	59 (36.4)	49 (37.1)	10 (33.3)	
University degree	38 (23.4)	31 (23.5)	7 (23.4)	
Marital status; *n* (%)				0.50
Single	16 (9.9)	12 (9.1)	4 (13.3)	
Married	146 (90.1)	120 (90.9)	26 (86.7)	
Chronic diseases; *n* (%)^b^				>0.99
Absent	118 (72.8)	96 (81.4)	22 (18.6)	
Present	44 (27.2)	36 (81.8)	8 (18.2)	
History of chronic diseases; *n* (%)				0.659
None	118 (72.8)	96 (72.7)	22 (73.3)	
Diabetes	15 (9.3)	11 (8.3)	4 (13.3)	
High blood pressure	8 (4.9)	6 (4.5)	2 (6.7)	
Heart disease	6 (3.7)	6 (4.5)	0 (0)	
Kidney disease	3 (1.9)	3 (2.3)	0 (0)	
Nervous system	3 (1.9)	3 (2.3)	0 (0)	
Asthma	2 (1.2)	1 (0.8)	1 (3.3)	
Other	7 (4.3)	6 (4.5)	1 (3.3)	
Signs and symptoms of COVID‐19 infection^a^; *n* (%)				0.026
Group 1	41 (25.3)	39 (29.5)	2 (6.7)	
Group 2	23 (14.2)	16 (12.1)	7 (23.3)	
Group 3	62 (38.3)	51 (38.6)	11 (36.7)	
Group 4	36 (22.2)	26 (19.7)	10 (33.3)	

^a^ Group 1: Fever + chills + shortness of breath and myalgia.

Group 2: Fever + shortness of breath + chills and headache.

Group 3: All symptoms.

Group 4: One of the symptoms such as fever, cough, headache, or shortness of breath.

^b^ Severity percentages is reported within having chronic diseases.

### 3.1 Alteration of sex hormones in female patients

Table 2 shows the hormone levels of patients at baseline and 1 month after discharge from the hospital. The estradiol level was 407.70 ± 623.37 pg/ml in patients with severe disease at baseline which reduced to 195.33 ± 380.04 pg/ml after discharge (*p* = 0.011). The level of estradiol was 213.78 ± 407.17 pg/ml in patients with moderate disease that reduced to 58.20 ± 39.45 pg/ml after discharge (*p* = 0.001).

**Table 2:** Hormone levels in hospitalized patients with severe and moderate infection upon hospitalization and 1 month after discharge

Hormones	Baseline	Follow‐up	*p* Value
Female patients			
Estradiol (pg/ml)			
Severe COVID‐19 infection	407.70 ± 623.37	195.33 ± 380.04	0.011
Moderate COVID‐19 infection	213.78 ± 407.17	58.20 ± 39.45	0.001
Progesterone (ng/ml)			
Severe COVID‐19 infection	2.03 ± 3.58	0.76 ± 0.52	0.440
Moderate COVID‐19 infection	1.14 ± 1.96	0.50 ± 0.29	0.140
LH (IU)			
Severe COVID‐19 infection	6.92 ± 3.73	5.96 ± 3.90	0.374
Moderate COVID‐19 infection	7.48 ± 8.16	7.01 ± 6.46	0.149
FSH (IU)			
Severe COVID‐19 infection	6.07 ± 3.68	5.55 ± 2.30	0.953
Moderate COVID‐19 infection	6.53 ± 7.98	6.16 ± 4.06	0.590
Male patients			
LH (IU)			
Severe COVID‐19 infection	6.64 ± 2.91	4.16 ± 2.44	<0.0001
Moderate COVID‐19 infection	6.42 ± 4.44	3.93 ± 3.15	0.102
FSH (IU)			
Severe COVID‐19 infection	3.98 ± 1.72	5.32 ± 2.55	0.140
Moderate COVID‐19 infection	4.53 ± 3.53	4.93 ± 2.58	0.446
Testosterone (ng/ml)			
Severe COVID‐19 infection	1.19 ± 0.73	2.64 ± 1.25	<0.0001
Moderate COVID‐19 infection	1.46 ± 1.22	2.54 ± 0.93	0.001
Free testosterone (ng/ml)			
Severe COVID‐19 infection	4.62 ± 3.12	4.80 ± 2.09	0.913
Moderate COVID‐19 infection	5.27 ± 2.90	4.72 ± 2.36	0.781

Abbreviations: FSH, follicle stimulating hormone; IU, international unit; LH, luteinizing hormone.

The level of progesterone was 2.03 ± 3.58 ng/ml in patients with severe disease at baseline that reduced to 0.76 ± 0.52 ng/ml 1 month after discharge. The alteration of this hormone in patients with moderate disease was negligible. In female patients, the level of LH was slightly high in both groups of patients with severe and moderate disease, but it reduced after discharge. Although the level of FSH was slightly high in patients with severe disease (6.07 ± 3.68 IU), it reduced to 5.55 ± 2.30 IU after discharge, but in patients with moderate disease, the alteration was negligible.

### 3.2 Alteration of sex hormones in male patients

In male patients, the level of LH in both groups of patients with severe (6.64 ± 2.91 IU) and moderate disease (6.42 ± 4.44 IU) was high, but it reduced after discharge from hospital (4.16 ± 2.44 and 3.93 ± 3.15, respectively). However, this decrease was significant only in patients with severe disease (*p* < 0.0001). The level of FSH was low at baseline in patients with severe disease but increased after discharge from the hospital.

The level of testosterone was 1.19 ± 0.73 and 1.46 ± 1.22 ng/ml at baseline in patients with severe and moderate disease, respectively, which increased to 2.64 ± 1.25 and 2.54 ± 0.93, respectively, *p* < 0.0001 and *p* = 0.001 after discharge. The alteration in free testosterone in both groups of severe and moderate patients was insignificant.

The relationship between severity of disease and the level of hormones was assessed using logistic regression, and the results are presented in Table 3. As evident from this table, there was no significant relationship between level of hormones and the severity of the disease.

**Table 3:** Relation between baseline hormone levels and severity of infection in hospitalized patients

Hormones	B (SE)	OR	95% CI	Wald	*p* Value
Female patients					
Estradiol (pg/ml)	0.001 (0.001)	1.001	(1.000−1.002)	1.573	0.210
Progesterone (ng/ml)	0.135 (0.122)	1.145	(0.902−1.454)	1.235	0.266
LH (IU)	−0.012 (0.051)	0.988	(0.894−1.092)	0.057	0.812
FSH (IU)	−0.012 (0.055)	0.988	(0.887−1.100)	0.047	0.828
Male patients					
LH (IU)	0.021 (0.061)	1.021	(0.905−1.152)	0.116	0.734
FSH (IU)	−0.055 (0.096)	0.946	(0.784−1.142)	0.331	0.565
Testosterone (ng/ml)	−0.245 (0.261)	0.783	(0.470−1.305)	0.880	0.348
Free testosterone (ng/ml)	−0.082 (0.088)	0.921	(0.775−1.094)	0.875	0.350

*Note*: Adjusted for age.

Abbreviations: CI, confidence interval; FSH, follicle stimulating hormone; IU, international unit; LH, luteinizing hormone; OR, odds ratio.

Table 4 shows the level of hormones in patients with and without chronic diseases. As evident from this table, the level of estradiol was significantly lower in female patients with chronic disease in follow‐up compared with those without chronic disease (122.37 ± 250.13 vs. 45.58 ± 45.63 ng/ml, *p* = 0.07). The LH level was significantly higher in female patients with chronic disease compared to those without chronic disease (10.35 ± 7.12 vs. 5.13 ± 4.39 IU, *p* = 0.042). The levels of progesterone and FSH were not significant between female patients with and without chronic diseases.

**Table 4:** Hormone levels in patients with and without chronic diseases at baseline and 1 month after discharge

Hormones	Baseline	Follow‐up	*p* Value
Female patients			
Estradiol (pg/ml)			
Without chronic disease	242.34 ± 443.67	122.37 ± 250.13	<0.0001
With chronic disease	224.30 ± 433.26	45.58 ± 45.63	0.011
Progesterone (ng/ml)			
Without chronic disease	1.03 ± 1.51	0.63 ± 0.44	0.162
With chronic disease	1.76 ± 3.32	0.45 ± 0.16	0.482
LH (IU)			
Without chronic disease	6.53 ± 5.74	5.13 ± 4.39	0.021
With chronic disease	9.55 ± 11.09	10.35 ± 7.12	0.767
FSH (IU)			
Without chronic disease	5.64 ± 5.10	5.41 ± 2.98	0.509
With chronic disease	8.48 ± 11.48	7.30 ± 4.67	0.859
Male patients			
LH (IU)			
Without chronic disease	6.45 ± 3.58	3.92 ± 2.44	0.001
With chronic disease	6.55 ± 5.47	4.44 ± 3.64	0.059
FSH (IU)			
Without chronic disease	4.40 ± 2.70	5.12 ± 2.34	0.304
With chronic disease	4.40 ± 4.39	5.21 ± 3.18	0.214
Testosterone (ng/ml)			
Without chronic disease	1.34 ± 1.00	2.70 ± 1.06	<0.0001
With chronic disease	1.55 ± 1.45	2.30 ± 1.20	0.022
Free testosterone (ng/ml)			
Without chronic disease	5.16 ± 2.91	4.85 ± 2.30	0.871
With chronic disease	4.97 ± 3.13	4.51 ± 1.90	0.799

Abbreviations: FSH, follicle stimulating hormone; IU, international unit; LH, luteinizing hormone.

Although the levels of testosterone and free testosterone were lower in male patients with chronic disease at baseline and 1 month after discharge, the differences were not significant.

No correlation was observed between duration of the disease and hormone levels (Table 5). One month after discharge, the most common symptom that persisted in patients was cough with a frequency of 76.7% and 89.7% in female and male patients, respectively (data are not shown in Tables).

**Table 5:** Relationship between hormone levels and disease duration in patients

Hormones	4 weeks or less	More than 4 weeks	*p* Value
Female patients			
Estradiol (pg/ml)	263.32 ± 455.51	89.68 ± 48.85	0.477
Median (Q_1_−Q_3_)	91 (65–149.3)	92.1 (51–137)	
Progesterone (ng/ml)	1.46 ± 2.76	3.07 ± 4.57	0.961
LH (IU)	8.52 ± 8.95	10.47 ± 9.43	0.573
FSH (IU)	7.00 ± 9.65	9.74 ± 11.67	0.351
Male patients			
LH (IU)	6.19 ± 2.85	5.82 ± 4.60	0.558
FSH (IU)	4.34 ± 2.37	3.58 ± 2.13	0.522
Testosterone (ng/ml)	1.25 ± 0.89	1.55 ± 0.86	0.341
Free testosterone (ng/ml)	4.60 ± 3.06	5.02 ± 3.57	0.714

Abbreviations: FSH, follicle stimulating hormone; LH, luteinizing hormone.

## 4 DISCUSSION

This study aimed to compare the level of sex hormones in hospitalized male and female patients with COVID‐19 upon hospitalization and 1 month after discharge. Our results indicated that the level of estradiol was significantly high in female patients with severe or moderate diseases during hospitalization, but it reduced after discharge. The level of progesterone was high during hospitalization among patients with severe disease but reduced after discharge, while in patients with moderate disease this alteration was negligible. Although the levels of LH and FSH increased at baseline and decreased after discharge, these alterations were not significant. Estrogen is well‐known to have a protective role in the women's body functions, especially against viral infections, and it has also been reported that this hormone can activate humoral immunity.^16^ Li et al.^11^ studied 237 female patients affected with COVID‐19 and found that patients with severe disease showed more irregularity in terms of menstrual volume and period, and were mainly characterized by decreased volume and prolonged cycles. Our results confirm those of Li et al. in that changes in the menstrual cycle can be due to hormonal imbalance in women. In a systematic review, Mikhail et al.^17^ showed that women with estradiol levels of more than 70 pg/ml were less likely to have severe disease, which is not consistent with our results that women with severe infection had higher levels of estrogen compared to those with moderate disease. This discrepancy may be attributed to the fact that the women recruited in the study of Mikhail et al., were premenopause, while our study involved women in their reproductive age.

The level of LH in male patients with severe and moderate disease was high and reduced after discharge. However, these changes were only significant in patients with severe infection. The level of FSH in male patients was low at baseline, and increased after discharge, but the changes were not significant. The level of testosterone was significantly low at baseline in both groups of patients with moderate and severe diseases, and it increased in the follow‐up. The alteration of free testosterone in male patients was negligible. The possible mechanism through which sex hormone alteration occurs in male patients affected with COVID‐19 is that the SARS‐CoV‐2 enters the cells via a receptor that called ACE2.^18^ High levels of ACE II have been reported in Leydig cells of testes that regulate testosterone via conversion of Angiotensin II into Angiotensin 1−7. Infection with COVID‐19 facilitates invasion of the virus to Leydig cells, causing alteration in male hormones.^19^ In line with our results, a systematic review on the level of male sex hormones showed that in patients with severe COVID‐19 infection, the level of total testosterone decreased significantly, and even men with lower levels of testosterone experienced more severe disease and mortality.^20^ Similar to our results, Dhindsa et al.^11^ in a study on 152 hospitalized patients found that men with severe disease had lower levels of testosterone in comparison to men with mild or moderate disease. Consistent with our results, Zeggeren et al.^21^ in a small study on 40 patients found that the level of total and free testosterone was significantly lower in patients with severe disease and in those who died from severe disease compared to survivors. By contrast, a case study carried out by Salama et al.^22^ and including 3 male cases with COVID‐19 infection found that while sexual function of male patients decreased, there was no alteration in the levels of total and free testosterone, LH, FSH, prolactin, and estradiol. Also, Xu et al.,^23^ in their study on 39 male patients affected with COVID‐19 found that the level of testosterone, FSH, LH, and prolactin did not alter significantly in patients with severe and moderate infection. The results of our study are not consistent with those of Salama et al. and Xu et al. This discrepancy may be due to the fact that the former was a case study and the latter study was conducted on a small sample of patients.

In addition to the role of hypothalamus in sex hormone alteration, the impact of mental disorders such as stress and depression on sex hormones cannot be ignored.^24^

In the present study, 34 out of 162 patients had severe disease and were transferred to the ICU. However, due to the small sample size and lack of sufficient studies in this area, we cannot draw a definitive conclusion about the relationship between sex hormone and severity of disease. In this study, we found that mortality rate is 5.5%, which is much higher than other reports from Iran.^25^ The higher mortality rate in the present study is due to the fact that in the present study we did not have patients with mild infection and all patients had moderate or severe disease.

The medications that the patients received in the hospital may have altered sex hormones. Patients in our study had received corticosteroid, anticoagulant medications, and antiviral drugs such as Remdesivir in the general wards or ICU.

### 4.1 Strengths and limitations of the study

This was the first study on sex hormones alteration during COVID‐19 pandemic in Iran, which makes the results of this study particularly valuable. Furthermore, we followed‐up the participants 1 month after discharge from hospital, which is another strength of this study. Despite its strengths, however, this study has some limitations that should be mentioned. In the present study, 162 patients with COVID‐19 infection were initially recruited of whom only 69 provided consent for blood sampling 1 month after discharge, and 9 patients died because of severity of the diseases. This rate of attrition may have affected the generalizability of the results. Furthermore, we did not record the menstrual cycle of women, and this might have had an important correlation with hormonal alteration in women. Therefore, the effect of COVID‐19 on sex hormones should be considered with caution.

## 5 CONCLUSION

Our findings showed that the level of estradiol in female patients increased significantly while the level of testosterone decreased in male patients during the active phase of infection. The changes in the progesterone, LH, and FSH in women were negligible. While the level of LH had an upward trend in male patients, their level of testosterone decreased significantly. Due to the loss of follow‐up for almost half of patients, our results should be considered with caution.

## AUTHOR CONTRIBUTIONS

**Poorandokht Afshari**: conceptualization; funding acquisition; methodology; supervision; writing – review & editing. **Mehrnoosh Zakerkish**: conceptualization; investigation; methodology; supervision; writing – review & editing. **Parvin Abedi**: conceptualization; investigation; methodology; software; supervision; validation; writing – original draft; writing – review & editing. **Maryam Beheshtinasab**: conceptualization; data curation; formal analysis; methodology; software; writing – review & editing. **Elham Maraghi**: conceptualization; data curation; formal analysis; methodology; software; writing – review & editing. **Hossein Meghdadi**: conceptualization; data curation; methodology; software; writing – review & editing.

## CONFLICT OF INTEREST

The authors declare no conflict of interest.

## ETHICS STATEMENT

This study was approved by the Ethics Committee of Ahvaz Jundishapur University of Medical Sciences, Ahvaz, Iran (Ref No: IR.AJUMS.REC.1399.541). All methods were performed in accordance with the relevant guidelines and regulations. All participants provided written informed consent before data collection.

## TRANSPARENCY STATEMENT

The lead author Parvin Abedi affirms that this manuscript is an honest, accurate, and transparent account of the study being reported; that no important aspects of the study have been omitted; and that any discrepancies from the study as planned (and, if relevant, registered) have been explained.

## Data Availability

The data sets generated and/or analyzed during the current study are not publicly available due to restrictions (Ahvaz Jundishapur University of Medical Sciences does not permit to data publicity before publication) but are accessible through the corresponding author upon reasonable request.
